# Evidence for Mixed Mg Coordination Environments in Silicate Glasses: Results from ^25^Mg NMR Spectroscopy at 35.2 T

**DOI:** 10.1021/acs.jpcb.3c06419

**Published:** 2023-11-30

**Authors:** Sabyasachi Sen, Jonathan F. Stebbins, Scott Kroeker, Ivan Hung, Zhehong Gan

**Affiliations:** † Department of Materials Science and Engineering, University of California at Davis, Davis, California 95616, United States; ‡ Department of Chemistry and Manitoba Institute for Materials, University of Manitoba, Winnipeg, Manitoba R3T 2N2, Canada; § Department of Earth and Planetary Sciences, Stanford University, Stanford, California 94305, United States; ∥ National High Magnetic Field Laboratory, 1800 East Paul Dirac Drive, Tallahassee, Florida 32310, United States

## Abstract

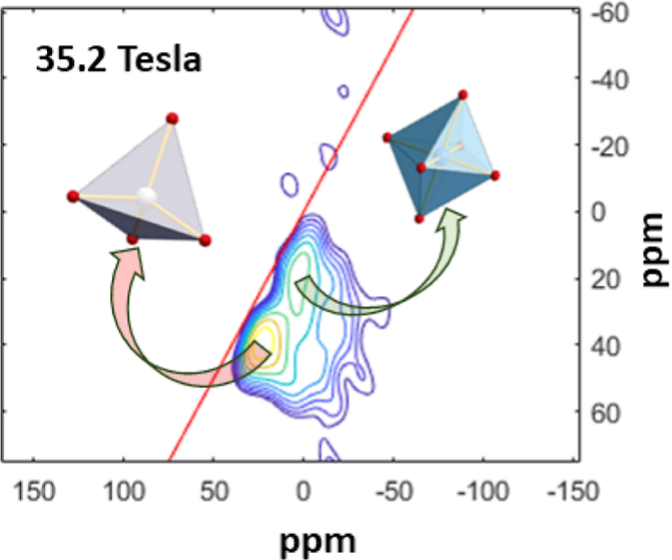

The Mg–O coordination environment of silicate glasses of composition CaMgSi_2_O_6_, Na_2_MgSi_3_O_8_, and K_2_MgSi_5_O_12_ is probed using ultrahigh-field (35.2 T) ^25^Mg magic angle spinning nuclear magnetic resonance (MAS NMR) and triple-quantum MAS NMR spectroscopy. These spectra clearly reveal the coexistence of 4-fold- (Mg^IV^) and 6-fold- (Mg^VI^) coordinated Mg in all glasses. The Mg^IV^/Mg^VI^ ratio implies an average Mg–O coordination number of ∼5 for CaMgSi_2_O_6_ glass, bringing NMR results for the first time in good agreement with those reported in previous studies based on diffraction and X-ray absorption spectroscopy, thus resolving a decade-long controversy regarding Mg coordination in alkaline-earth silicate glasses. The Mg–O coordination number decreases to ∼4.5 in the alkali–Mg silicate glasses, indicating that Mg competes effectively with the low field strength alkali cations for the nonbridging oxygen in the structure to attain tetrahedral coordination. This work illustrates the promise of ultrahigh-field NMR spectroscopy in structural studies involving nuclides with low gyromagnetic ratio.

## 1 Introduction

Recent studies have indicated that Mg plays a key role as a constituent element in silicate glasses in improving their crack and corrosion resistance as well as certain aspects of their bioactivity.^1,2^ Moreover, Mg is an important geochemical constituent in many natural silicate melts or magmas that exerts significant control on the transport properties and mineral–melt equilibria.^3−5^ These functional roles of Mg are critically dependent on its bonding and coordination environment in the structures of silicate glasses and liquids. On one hand, it is well-established that the addition of MgO to a silicate glass results in the formation of nonbridging oxygen atoms (NBOs). In that sense, it can be argued that MgO plays the role of a modifier oxide.^6^ On the other hand, increasing addition of MgO to SiO_2_ beyond 50 mol % results in an increase in the glass transition temperature, which could be suggestive of the participation of strong Mg–O bonds in the integrity of the structural network.^7^

However, despite a large number of structural studies utilizing a variety of spectroscopic and diffraction methods, the nature of the Mg–O coordination environment in silicate glasses has remained rather controversial in the literature. For example, previous X-ray absorption spectroscopic studies at the Mg K-edge indicated the presence of Mg^IV^ (tetrahedral or 4-fold coordination), Mg^V^ (5-fold coordination), and Mg^VI^ (octahedral or 6-fold coordination) environments in the structure of CaMgSi_2_O_6_ glass.^8,9^ On the other hand, ^25^Mg magic angle spinning nuclear magnetic resonance (MAS NMR) studies conducted at a magnetic field of 14.1 T by Kroeker and Stebbins indicated that, like crystalline CaMgSi_2_O_6_, Mg is present predominantly as Mg^VI^ in the structure of the glass of the same composition.^10^ The same conclusion was reached by Sen et al. for glasses along the join Mg_2_SiO_4_–MgSiO_3_ based on ^25^Mg MAS NMR spectra acquired at a magnetic field of 21.8 T and by Shimoda et al. for MgSiO_3_, CaMgSi_2_O_6_, and a few other Ca, Mg silicate and aluminosilicate glasses on the basis of ^25^Mg triple-quantum MAS (3QMAS) NMR studies at 16.4 and 21.8 T.^6,11,12^ These results on the Mg coordination environment in MgSiO_3_ and CaMgSi_2_O_6_ glasses have recently been corroborated in a ^25^Mg NMR study conducted at 14.1 T by Eckert and co-workers;^13^ but this study also indicated that the presence of multiple Mg coordination environments in these glasses cannot be precluded solely on the basis of NMR data at these moderate magnetic fields. In contrast, isotope-substituted neutron diffraction studies by Cormier and Cuello and combined X-ray and neutron diffraction and reverse Monte Carlo simulation by Wilding et al. indicated that Mg is present predominantly as Mg^IV^ in MgSiO_3_ and other MgO–SiO_2_ glasses, with an average Mg–O coordination number ranging between 4.4 and 4.8.^7,14,15^

Such a significant discrepancy between diffraction and NMR results may be indicative of the challenges associated with each technique for the determination of the coordination environment of cations, such as Mg. While, for diffraction, a distorted nearest-neighbor environment may lead to an erroneous estimation of the coordination number of an atom, NMR spectroscopy of the ^25^Mg nuclide presents its own formidable challenges owing to the low gyromagnetic ratio of this nuclide, combined with its low natural abundance (10%) and large quadrupolar broadening. These challenges with ^25^Mg NMR can be circumvented by isotopically enriching the samples with ^25^Mg and by performing MAS and multiple-quantum MAS (MQMAS) NMR spectroscopy at ultrahigh magnetic fields (>20 T) to minimize or eliminate the effects of quadrupolar broadening. The MQMAS experiment is a two-dimensional NMR spectroscopic technique that averages out the second-order line-broadening effects from quadrupolar interactions for *I* > 1/2 nuclides such as ^25^Mg (*I* = 5/2), by mixing single- and multiple-quantum coherences to generate a high-resolution isotropic spectrum in one dimension, which is correlated in the second dimension with the single-quantum MAS central-transition spectrum.^16^ The isotropic spectrum is only broadened by the distributions of the chemical shift and the isotropic quadrupolar shift. Here we present high-resolution ^25^Mg MAS and 3QMAS NMR spectroscopic data on select crystalline and glassy alkali and alkaline-earth Mg silicates obtained at an ultrahigh magnetic field of 35.2 T. These spectra reveal the Mg coordination environment in these materials to unprecedented accuracy and conclusively demonstrate the coexistence of multiple Mg–O coordination environments in silicate glasses, which resolves the above-mentioned inconsistency between the NMR and the diffraction results reported in previous studies in the literature.

## 2 Materials and Methods

### 2.1 Materials

The crystalline CaMgSi_2_O_6_ (diopside), Ca_2_MgSi_2_O_7_ (akermanite), and K_2_MgSi_5_O_12_ and glassy CaMgSi_2_O_6_, Na_2_MgSi_3_O_8_, and K_2_MgSi_5_O_12_ samples used in this study are the same ones used in a previous ^25^Mg NMR study by Kroeker and Stebbins,^10^ except the glass samples were all rejuvenated for this study before the NMR data collection by melting them at 1450 °C for 1 h in a platinum crucible followed by quenching in air. All samples were originally prepared by Kroeker and Stebbins using 96.75% ^25^Mg-enriched MgO.^10^ The crystalline compounds were prepared via solid-state synthesis, and their phase identification was carried out using powder X-ray diffraction. The glasses were prepared by using the typical melt-quenching route. Details of the sample synthesis can be found in that study.

### 2.2 Ultrahigh-Field ^25^Mg NMR

^25^Mg solid-state NMR experiments recorded at 35.2 T were performed on the series-connected hybrid (SCH) magnet (^25^Mg Larmor frequency of 91.83 MHz) at the National High Magnetic Field Laboratory (NHMFL) in Tallahassee, USA.^17^ A Bruker AVANCE NEO console and a single-resonance MAS probe designed and constructed at the NHMFL were used with 3.2 mm o.d. pencil-style rotors spinning at a MAS frequency of 17.86 kHz. The 1D NMR spectra for the crystalline CaMgSi_2_O_6_ (diopside), Ca_2_MgSi_2_O_7_ (akermanite), and K_2_MgSi_5_O_12_ samples were obtained using a single-pulse direct excitation of 2 μs, *rf* field of 14.9 kHz, recycle delay of 1 s, and 1024 averaged transients. For the CaMgSi_2_O_6_, K_2_MgSi_5_O_12_, and Na_2_MgSi_3_O_8_ glass samples, the 1D NMR spectra were acquired using QCPMG^18^ signal enhancement with π/2 and π pulses of 3 and 6 μs, 0.5 s recycle delay, 54 CPMG pulse acquisition loops with each loop lasting 20 rotor periods, and 3840, 5120, and 8192 averaged transients, respectively. A phase-modulated split-*t*_1_ shifted-echo sequence^19^ was used for acquisition of the 2D 3QMAS NMR spectra in combination with QCPMG for signal enhancement. Pulses of 9.9 and 3.3 μs with *rf* field of ∼44 kHz were used for 3Q excitation and conversion, along with soft pulses of 5 and 10 μs at 16.7 kHz *rf* field, 0.5 s recycle delay, and 68 CPMG pulse-acquisition loops, with each loop lasting 20 rotor periods. Acquisition of the *t*_1_ evolution was performed by rotor synchronization of the delay between the 3Q excitation and conversion pulses; 8 complex *t*_1_ points were acquired for each spectrum with a total of 2880, 8640, and 5760 and averaged transients per *t*_1_ point for the CaMgSi_2_O6, K_2_MgSi_5_O_12_, and Na_2_MgSi_3_O_8_ glass samples, respectively. ^25^Mg NMR spectra were externally referenced by recording the ^17^O signal of D_2_O and using the ^17^O and ^25^Mg frequency ratios reported in the IUPAC recommendations.^20^

## 3 Results and Discussion

**Figure 1:**
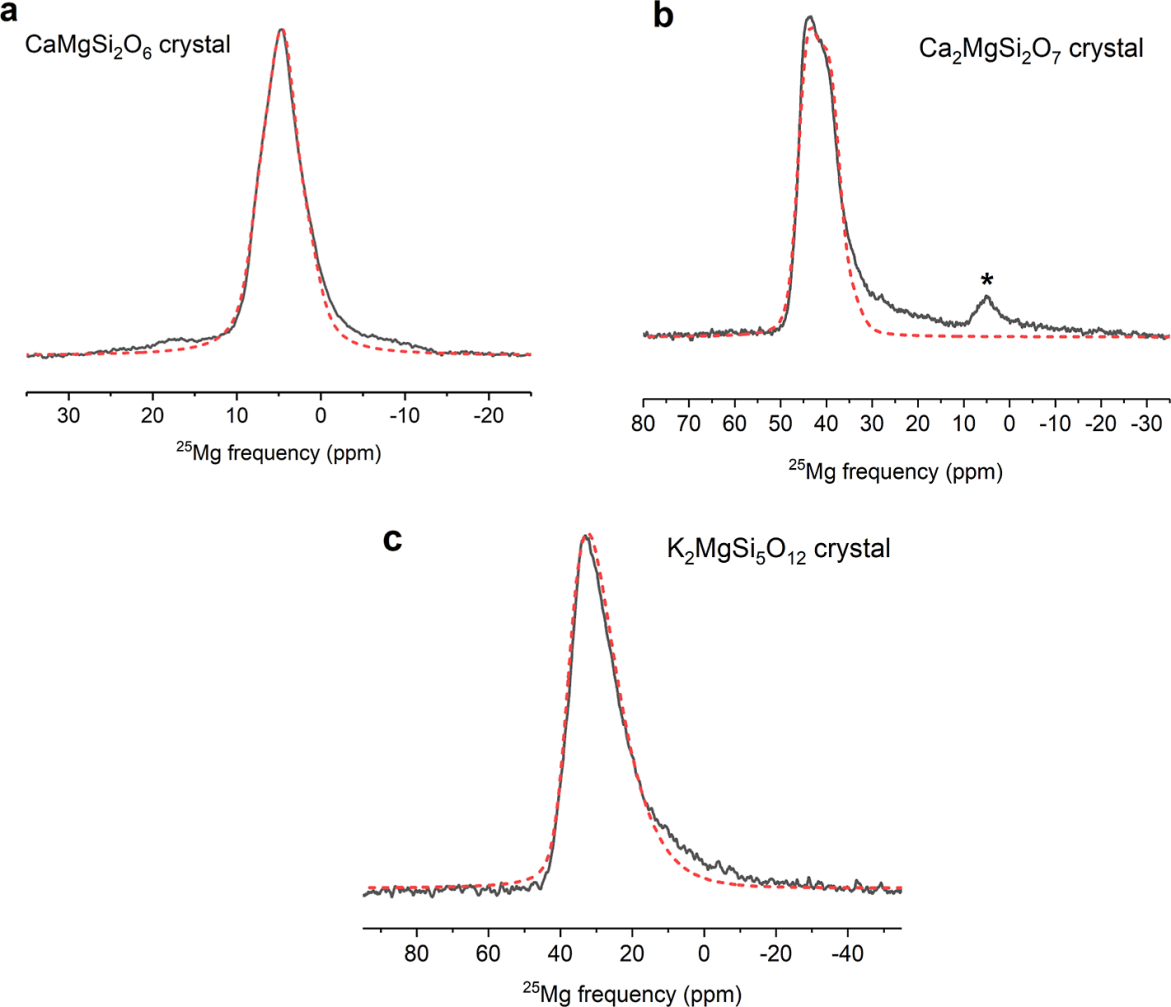
Experimental (solid black line) and simulated (dashed red line) ultrahigh-field (35.2 T) ^25^Mg MAS NMR spectra of crystalline silicates: (a) CaMgSi_2_O_6_, (b) Ca_2_MgSi_2_O_7_, and (c) K_2_MgSi_5_O_12_. Resonance marked by an asterisk in (b) corresponds to the CaMgSi_2_O_6_ secondary phase. Simulation parameters are listed in Table 1.

The ultrahigh-field ^25^Mg MAS NMR spectra of the three crystalline compounds CaMgSi_2_O_6_ (diopside), Ca_2_MgSi_2_O_7_ (akermanite), and K_2_MgSi_5_O_12_ are shown in Figure 1. The center bands of these central-transition line shapes were simulated using the ssNake software package to obtain the NMR parameters including the isotropic chemical shift δ_iso_, the quadrupolar coupling constant *C*_Q_, and the asymmetry parameter η of the electric field gradient tensor at the site of the nucleus.^21^ As shown in Table 1, the resulting NMR parameters are consistent with those obtained in a previous study by Kroeker and Stebbins from simulation of low-field (14.1 T) line shapes on the same samples.^10^ It is apparent from Figure 1 that while the central-transition MAS line shapes of crystalline CaMgSi_2_O_6_ and Ca_2_MgSi_2_O_7_ can be described well with a single set of δ_iso_, *C*_Q_, and η, that of crystalline K_2_MgSi_5_O_12_ requires a distribution of quadrupolar parameters. This difference was explained by Kroeker and Stebbins^10^ to be due to a disordered structure of the K_2_MgSi_5_O_12_ sample, owing to its synthesis conditions. An extended Czjzek distribution of the quadrupolar parameters^21,22^ was therefore employed within the ssNake program to simulate the ^25^Mg NMR line shape for this crystal. The Mg atoms are known to be octahedrally coordinated in crystalline CaMgSi_2_O_6_, and tetrahedrally coordinated in K_2_MgSi_5_O_12_ and Ca_2_MgSi_2_O_7_, consistent with their ^25^Mg NMR δ_iso_ values of ∼8, 39, and 47 ppm, respectively, as obtained from these simulations (Table 1). It may be noted here that in the present study, the significant lowering of the quadrupolar broadening in the ultrahigh-field ^25^Mg MAS NMR spectra of these crystals reveals the presence of a small amount (∼4%) of a secondary “impurity” phase in the Ca_2_MgSi_2_O_7_ sample in the form of a resonance centered near ∼5 ppm (Figure 1). The presence of this phase went undetected in the lower-field ^25^Mg MAS NMR spectrum of this sample in the previous study by Kroeker and Stebbins.^10^ Moreover, the small fraction of this phase must have remained undetected in their powder X-ray diffraction measurement as well. The spectral line shape of this resonance could indeed be simulated with a set of NMR δ_iso_, C_Q_, and η that is consistent with this secondary phase being crystalline CaMgSi_2_O_6_.

**Table 1:** ^25^Mg NMR Parameters for Crystalline Compounds Obtained from Simulation of Spectra Acquired at 35.2 T^a^

composition	δ_iso_ (±0.5 ppm)	*C*_Q_ (±0.1 MHz)	η (±0.05)
Ca_2_MgSi_2_O_7_ (akermanite)	47.4 (47.0)	2.9 (2.8)	0.23 (0.20)
CaMgSi_2_O_6_ (diopside)	8.4 (8.0)	2.1 (2.1)	0.75 (0.75)
K_2_MgSi_5_O_12_	39.2 (30–50)	3.1 (<3.7)	0.00

a Values in parentheses are from ref (10).

**Figure 2:**
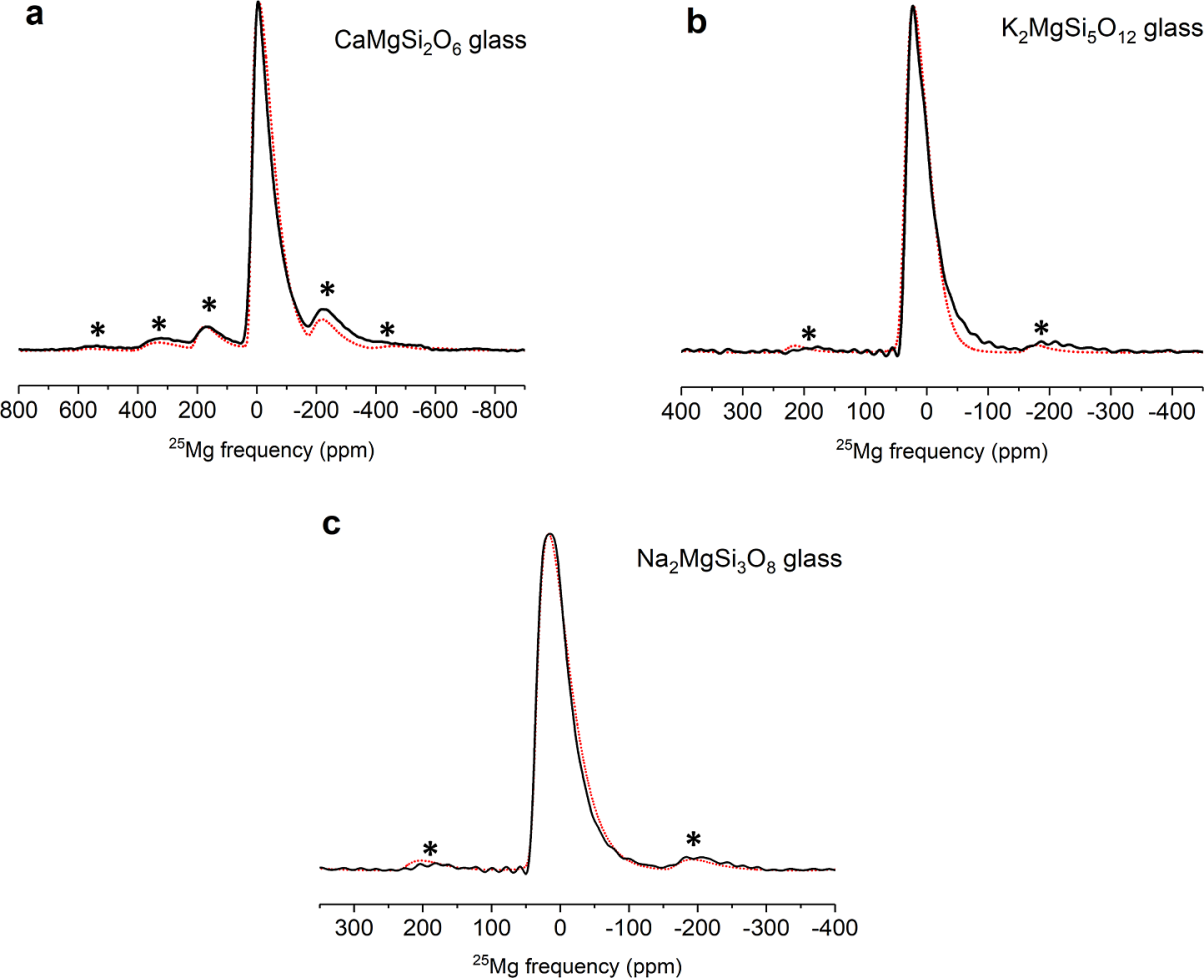
Experimental (solid black line) and simulated (dotted red line) ultrahigh-field (35.2 T) ^25^Mg MAS NMR spectra of glassy silicates: (a) CaMgSi_2_O_6_, (b) K_2_MgSi_5_O_12_, and (c) Na_2_MgSi_3_O_8_. Asterisks denote spinning sidebands. Simulation parameters are listed in Table 2. Note different ppm scales for the spectra.

The ultrahigh-field ^25^Mg MAS NMR spectra of the three glasses (CaMgSi_2_O_6_, K_2_MgSi_5_O_12_, and Na_2_MgSi_3_O_8_) investigated in this study are shown in Figure 2. These spectral line shapes can be simulated well, with a single site having an extended Czjzek distribution of quadrupolar parameters (Table 2). A direct comparison between the ^25^Mg NMR parameters (Tables 1 and 2) of the crystalline and glassy phases of CaMgSi_2_O_6_ and K_2_MgSi_5_O_12_ shows relatively small change in δ_iso_ between the crystal and the glass, suggesting that Mg is predominantly 6-fold (4-fold)-coordinated in CaMgSi_2_O_6_ (K_2_MgSi_5_O_12_) glass. On the other hand, as expected, the average C_Q_ for the ^25^Mg MAS NMR line shape increases by nearly 4× in the CaMgSi_2_O_6_ glass and by 2× in the K_2_MgSi_5_O_12_ glass, with respect to their crystalline counterparts (Tables 1 and 2), which can be attributed to a corresponding increase in the structural disorder of the Mg coordination environment. Finally, a strong similarity between the ^25^Mg NMR parameters of K_2_MgSi_5_O_12_ and Na_2_MgSi_3_O_8_ glass indicates a corresponding similarity in their Mg coordination environments (Table 2). These ^25^Mg MAS NMR results and the general conclusions obtained at the ultrahigh magnetic field of 35.2 T are completely consistent with those obtained in previous studies carried out at 14.1 T, and besides validating each other they do not provide any new information.^10,13^ However, as discussed below, a significantly different structural scenario emerges when the results of the ultrahigh-field ^25^Mg 3QMAS NMR spectra of these glasses are considered.

**Table 2:** ^25^Mg NMR Parameters for Glasses Obtained from Simulation of Spectra Acquired at 35.2 T Using a Single Site with an Extended Czjzek Distribution^a^

composition	δ_iso_ (±2 ppm)	average *C*_Q_ (±0.1 MHz)^b^	width of Czjzek distribution σ (±0.1 MHz)	average η^b^
CaMgSi_2_O_6_	16	7.8	2.6	0.0
K_2_MgSi_5_O_12_	38	6.3	1.2	0.0
Na_2_MgSi_3_O_8_	36	6.3	2.0	0.0

a A Gaussian line-broadening of 10 ppm was used to account for a distribution of isotropic chemical shifts. The mean value of η for the distribution was kept constant for all simulations.

b Mean value of the extended Czjzek distribution.

**Figure 3:**
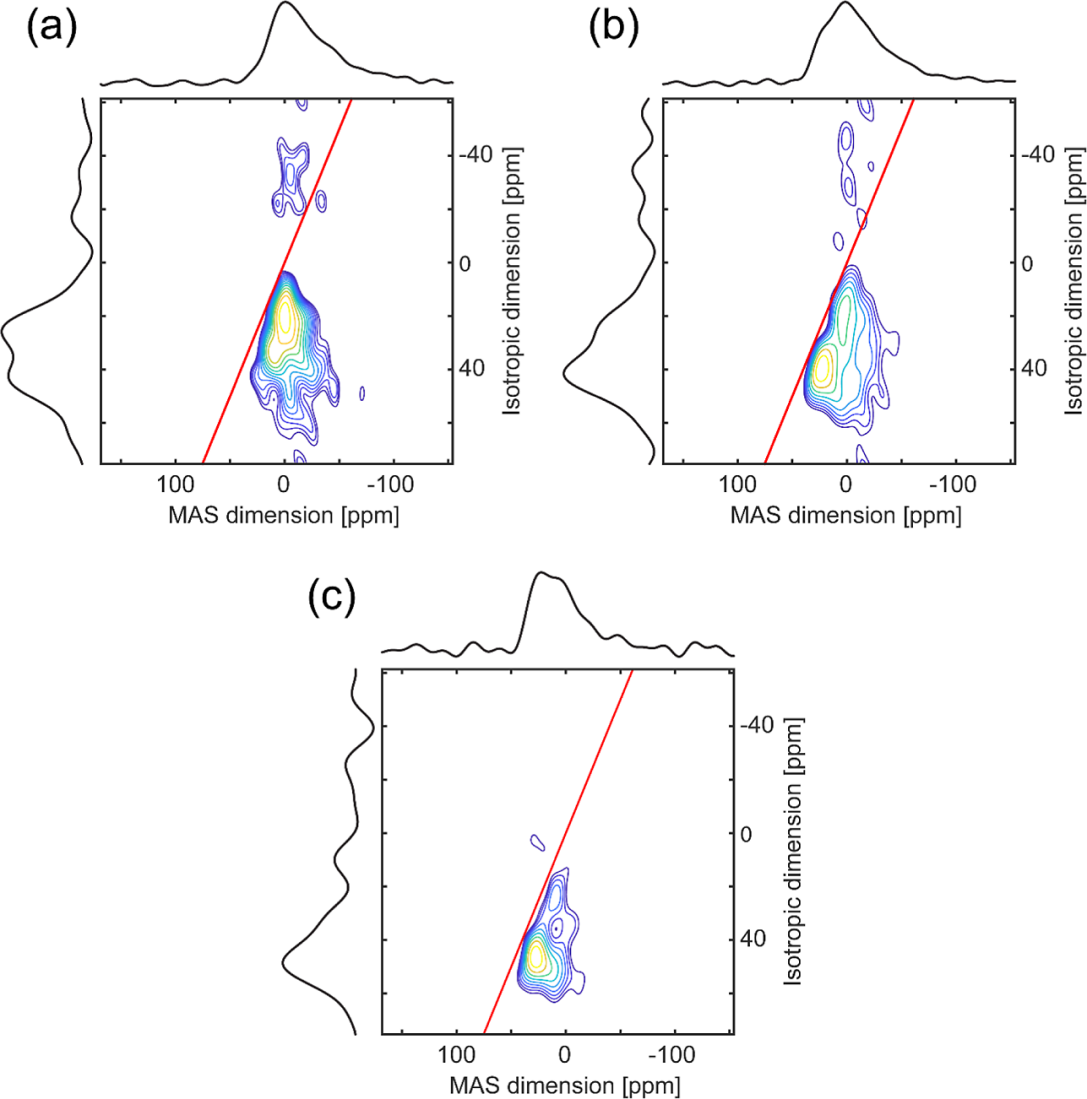
Contour plots of ^25^Mg 3QMAS NMR spectra of (a) CaMgSi_2_O_6_, (b) K_2_MgSi_5_O_12_, and (c) Na_2_MgSi_3_O_8_ glasses. Total projections on isotropic and MAS dimensions are shown on the left and top. The red straight line across each plot denotes a quadrupolar-induced shift.

The ^25^Mg 3QMAS NMR spectra of the CaMgSi_2_O_6_, K_2_MgSi_5_O_12_, and Na_2_MgSi_3_O_8_ glasses are shown in Figure 3. The total projection of these spectra along the isotropic dimension resolves the presence of at least two Mg resonances in all three cases. For a spin *I* = 5/2 nuclide such as ^25^Mg, the δ_iso_ and the quadrupolar product *P*_Q_ = *C*_Q_ (1 + η^2^/3) for the two resonances can be estimated from their isotropic peak position δ_*F*1_ and their center of gravity δ_*F*2_^CG^ along the MAS dimension using the following relations^23^
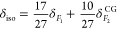
1
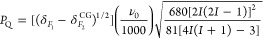
2where ν_0_ is the resonance frequency (91.83 MHz) and *I* = 5/2 is the spin quantum number of the nuclide. Such calculations yield δ_iso_ and *P*_Q_ values ranging between 12 and 19 ppm and 3.3 and 5.5 MHz, respectively, for one and 30 and 40 ppm and 5.0 and 7.0 MHz, respectively, for the other ^25^Mg NMR resonance (Table 3). Unfortunately, the structural assignment of these ^25^Mg resonances on the basis of their δ_iso_ values is not an entirely straightforward task due to the fact that high-resolution ^25^Mg NMR measurements on well-characterized crystalline compounds remain rather limited in the literature to establish clear chemical shift ranges for specific Mg–O coordination environments in silicates. Previous ^25^Mg NMR studies^11,24,25^ indicated that the δ_iso_ for Mg^VI^ environments in some silicate minerals ranged between ∼1 and 15 ppm, while the results of the present study on crystalline silicates as listed in Table 1 indicate that the δ_iso_ for Mg^IV^ environments can at least be as low as ∼39 ppm. Therefore, the two resonances with low (12–19 ppm) and high (30–40 ppm) δ_iso_ values in the ^25^Mg 3QMAS NMR spectra of CaMgSi_2_O_6_, K_2_MgSi_5_O_12_, and Na_2_MgSi_3_O_8_ glasses can be tentatively assigned to the Mg^VI^ and Mg^IV^ environments, respectively. However, the validity of this assignment hinges somewhat upon whether a fraction of Mg atoms in the structure of these glasses can also be present in 5-fold coordination with oxygen, i.e., as Mg^V^. Unfortunately, the characteristic δ_iso_ for Mg^V^ species remains unknown at present, though it is expected to lie between ∼20 and 30 ppm (see below). Nevertheless, the existence of Mg^V^ species in these glasses cannot be precluded solely on the basis of the NMR data presented in this study.

**Table 3:** ^25^Mg NMR Parameters for Glasses Obtained from Simulation of 1D ^25^Mg MAS Spectra Acquired at 35.2 T Using Two Sites with Extended Czjzek Distributions^a^

composition	sites	δ_iso_ (±2 ppm)	average *C*_Q_ (±0.1 MHz)^b^	width of Czjzek distribution σ (±0.1 MHz)	average η^b^	relative fraction (%)
CaMgSi_2_O_6_	Mg^IV^	30 (30)	7.7 (7.0)	3.0	0.0	44
	Mg^VI^	16 (12)	6.0 (5.0)	3.0	0.0	56
K_2_MgSi_5_O_12_	Mg^IV^	39 (34)	5.5 (6.0)	0.8	0.0	70
	Mg^VI^	12 (15)	6.0 (5.5)	2.5	0.0	30
Na_2_MgSi_3_O_8_	Mg^IV^	38 (40)	5.1 (5.0)	2.0	0.0	75
	Mg^VI^	16 (19)	4.0 (3.3)	3.0	0.0	25

a A Gaussian line-broadening of 10 ppm was used to account for a distribution of isotropic chemical shifts. The mean value of η for the distribution was kept constant for all simulations. Values in parentheses are δ_iso_ and P_Q_ obtained from peak positions in F1 and center of gravity positions in F2 dimensions of the 3QMAS spectra using eqs 1 and 2.

b Mean value of the extended Czjzek distribution.

**Figure 4:**
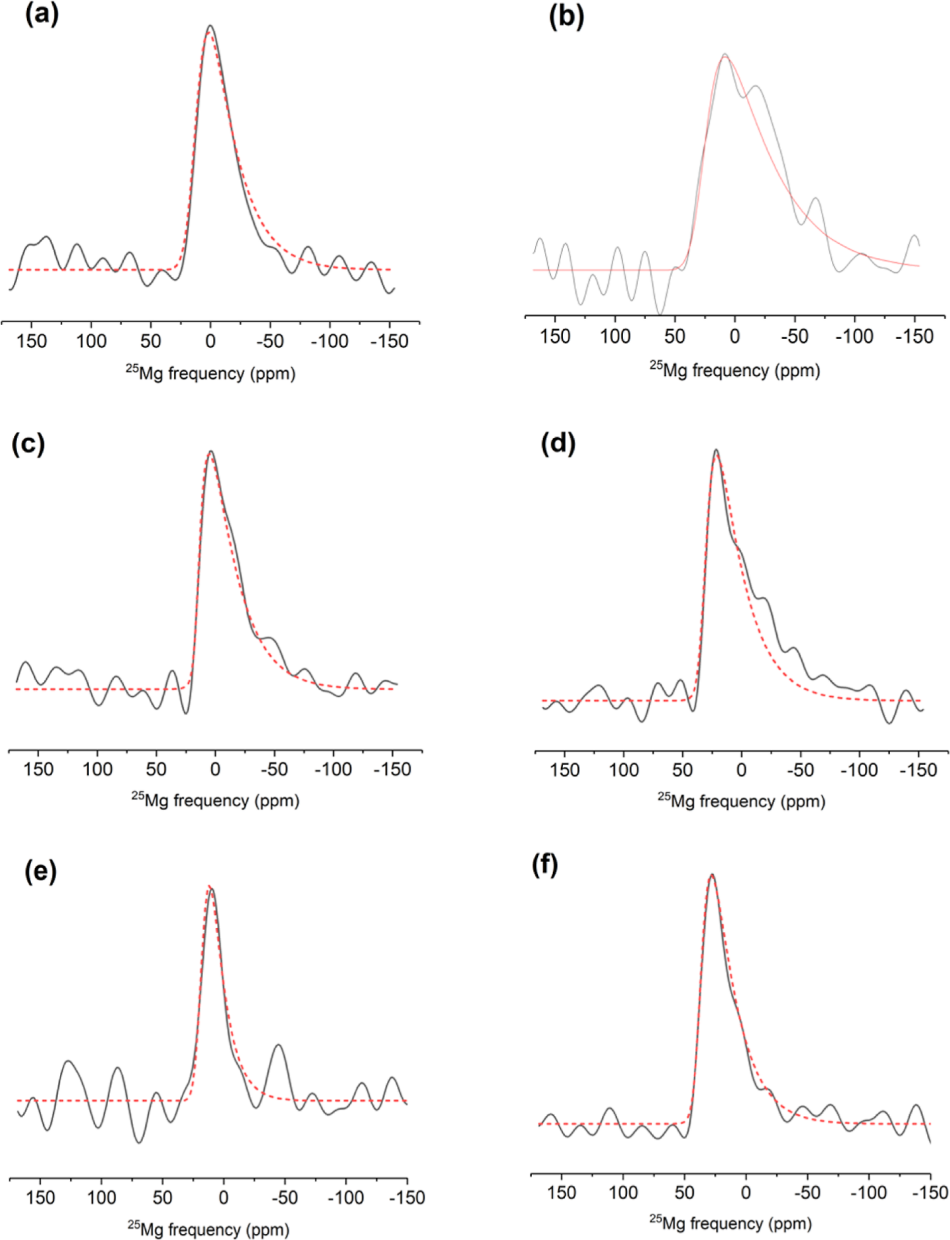
MAS slices of ^25^Mg 3QMAS spectra at the two isotropic peak positions (solid lines) and corresponding simulations (dashed lines) for CaMgSi_2_O_6_, K_2_MgSi_5_O_12_, and Na_2_MgSi_3_O_8_ glasses. MAS slices are taken at isotropic peak positions of (a) 14 and (b) 38 ppm for CaMgSi_2_O_6_ glass; (c) 20 and (d) 40 ppm for K_2_MgSi_5_O_12_ glass; and (e) 25 and (f) 47 ppm for Na_2_MgSi_3_O_8_ glass.

**Figure 5:**
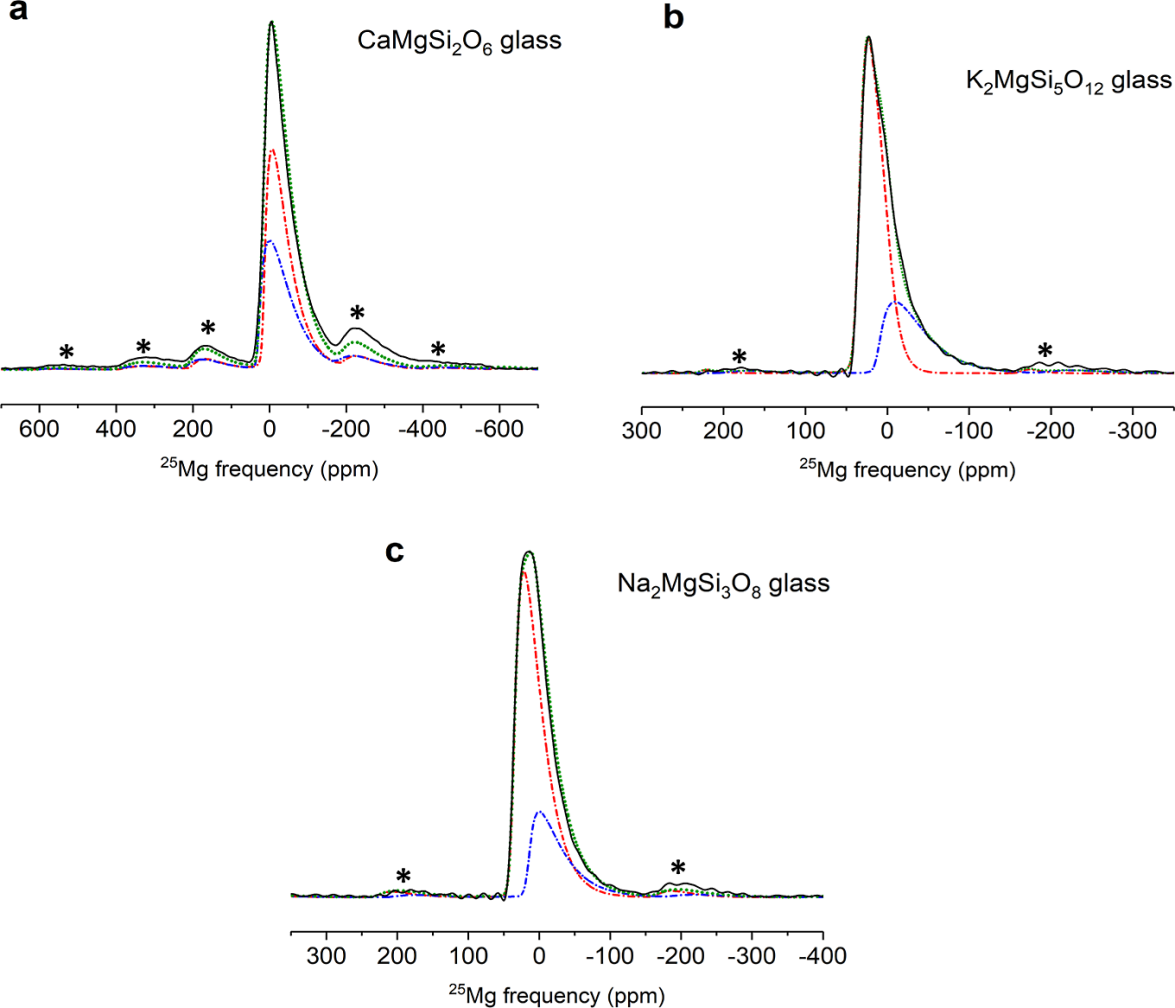
Two-component simulation of experimental 1D ^25^Mg MAS NMR line shapes (black solid line) for (a) CaMgSi_2_O_6_, (b) K_2_MgSi_5_O_12_, and (c) Na_2_MgSi_3_O_8_ glasses. Simulation is shown as a green dotted line and individual components are shown with red and blue dot-dashed lines. Asterisks denote spinning sidebands. Note different ppm scales for the spectra.

It may be noted here that in the absence of any constraint on η, the *P*_Q_ values obtained from the peak positions in the 3QMAS spectra bracket the estimate of *C*_Q_ to within ±15%. A second estimate of δ_iso_ and *C*_Q_ for these two resonances can be obtained from simulations of the MAS projections of these 3QMAS spectra at the two isotropic peak positions using a Czjzek distribution. These simulations are shown in Figure 4, which yield average δ_iso_ and *C*_Q_ values for the Mg^VI^ and Mg^IV^ environments in these glasses that are consistent with the estimates obtained from the 3QMAS spectral peak positions. To ensure further consistency, these average δ_iso_ and *C*_Q_ values for the three glasses are used as initial guesses along with an extended Czjzek distribution of the quadrupolar parameters to simulate their 1D ^25^Mg MAS NMR line shapes in Figure 2. It is clear from Figure 5 that such two-component simulations are indeed able to reproduce the 1D MAS NMR line shapes quite well. The peak areas obtained from these simulations provide the relative fraction of the Mg^VI^ and Mg^IV^ environments in these glasses (Table 3). This procedure yields a Mg^VI^/Mg^IV^ ratio of 56:44 for the CaMgSi_2_O_6_ glass. This ratio decreases significantly to 30:70 for the K_2_MgSi_5_O_12_ glass and 25:75 for the Na_2_MgSi_3_O_8_ glass. These ratios are found to be completely consistent with those estimated from a simulation of the total isotropic projection spectra with Gaussian components under the assumption of uniform triple-quantum excitation for both Mg environments.

The resulting average Mg–O coordination number of ∼5.1 for the CaMgSi_2_O_6_ glass is consistent with that (∼5.0) reported on this composition by Ildefonse et al.^8^ on the basis of a Mg K-edge extended X-ray absorption fine structure (EXAFS) spectroscopic study. Moreover, the simulation parameters in Table 3 yield an average ^25^Mg δ_iso_ of ∼22 ppm, in excellent agreement with the same value reported by George and Stebbins^26^ for the CaMgSi_2_O_6_ liquid using high-temperature ^25^Mg NMR spectroscopy, lending further support to the Mg–O coordination number obtained in the present study. This Mg–O coordination number is also consistent with that (∼5.0) obtained in a recent study by Cormier and Cuello^27^ for glass of composition Ca_1.5_Mg_0.5_Si_2_O_6_ using combined X-ray and neutron diffraction and reverse Monte Carlo simulation. When taken together, this agreement between NMR, EXAFS, and diffraction studies resolves the long-standing inconsistency between the Mg–O coordination numbers for silicate glasses reported in previous studies and the related controversy in the literature, as noted earlier. It is expected that a similar situation will also hold for the MgSiO_3_ glass as diffraction studies have indicated that the average Mg–O coordination number for this glass is lower (∼4.5) compared to its Ca-rich analogues, and this coordination number monotonically decreases with increasing Mg/Ca in glasses along the binary join CaSiO_3_–MgSiO_3_.^14,15,27,28^

In a previous study, Kroeker and Stebbins hypothesized that Mg competes more effectively with the low field strength alkali cations than with the higher field strength Ca for the NBOs in the silicate network to form more compact tetrahedral coordination environments. This hypothesis is corroborated by the ^25^Mg 3QMAS results, which clearly show that compared to the CaMgSi_2_O_6_ glass, the Mg^VI^/Mg^IV^ ratio is significantly lowered in the alkali–Mg silicate glasses (Table 3). It may be noted that a lowering of the average Mg–O coordination number with increasing Na/Mg ratio was also reported in a previous study by Bisbrouck et al.^29^ for Na–Mg boroaluminosilcate glasses and a predominantly tetrahedral Mg–O coordination was suggested by Shimoda et al. for Na–Mg and K–Mg silicate glass.^11^ The results of the present study indicate that the average Mg–O coordination number is ∼4.6 for the K_2_MgSi_5_O_12_ glass and ∼4.5 for the Na_2_MgSi_3_O_8_ glass (Table 3). The average ^25^Mg δ_iso_ of ∼31–32 ppm for these glasses (Table 3) is again in good agreement with those (∼27–30 ppm) reported by George and Stebbins^26^ for Na–Mg and Na, K–Mg silicate liquids using high-temperature ^25^Mg NMR spectroscopy. It may be noted here that the tetrahedral coordination of Mg does not necessarily mean that a part of the Mg ions play the role of network-forming cations in these glasses. This is particularly evident in the ^29^Si and ^17^O NMR spectra of glasses of similar compositions reported in the literature, which indicate that the degree of Si–O network connectivity remains consistent with Mg acting as a network modifier in these glasses.^30−32^

## 4 Conclusions

In summary, a combination of ^25^Mg MAS and 3QMAS NMR at an ultrahigh magnetic field enables identification of mixed Mg–O coordination environments in silicate glass. The results are interpreted in terms of the coexistence of Mg^IV^ and Mg^VI^ environments in all glasses, although the possibility of the presence of some fraction of Mg in a Mg^V^ environment cannot be ruled out. The relative ratios of Mg^IV^ and Mg^VI^, thus obtained for CaMgSi_2_O_6_ glass, yield an average Mg–O coordination number of ∼5, which is in good agreement with the results obtained in previous Mg K-edge EXAFS and diffraction measurements. This agreement resolves a long-standing inconsistency between Mg–O coordination numbers in silicate glasses derived via NMR spectroscopy vis-à-vis X-ray absorption spectroscopy and diffraction techniques. When taken together, the ^25^Mg δ_iso_ values for various silicate crystals, glasses, and liquids obtained in this study and in previous studies in the literature allow for the establishment of ^25^Mg chemical shift ranges of ∼5–17, 20–30, and 30–50 ppm, for Mg^IV^, Mg^V^, and Mg^VI^ environments, respectively. Mg shows a stronger preference for tetrahedral coordination in alkali–Mg silicate glasses compared to CaMgSi_2_O_6_ glass, implying that Mg competes for NBO more effectively with low field strength alkali ions than with higher field strength Ca ions. The average ^25^Mg δ_iso_ values for all glasses are found to be in good agreement with those reported for liquids of similar composition in previous high-temperature ^25^Mg NMR spectroscopic studies.
